# Properties of monocytes generated from haematopoietic CD34^+^ stem cells from bone marrow of colon cancer patients

**DOI:** 10.1007/s00262-012-1375-5

**Published:** 2012-11-24

**Authors:** Malgorzata Stec, Jarosław Baran, Rafał Szatanek, Bożenna Mytar, Marzena Lenart, Antoni Czupryna, Antoni Szczepanik, Maciej Siedlar, Marek Zembala

**Affiliations:** 1 Department of Clinical Immunology and Transplantation, Polish-American Institute of Paediatrics, Jagiellonian University Medical College, Wielicka str. 265, 30-663 Cracow, Poland; 2 First Department of General and Gastrointestinal Surgery, Jagiellonian University Medical College, Cracow, Poland

**Keywords:** Cancer patients, Bone marrow, CD34^+^ stem cells, Monocyte subpopulations, Tumour cells

## Abstract

Monocytes exhibit direct and indirect antitumour activities and may be potentially useful for various forms of adoptive cellular immunotherapy of cancer. However, blood is a limited source of them. This study explored whether monocytes can be obtained from bone marrow haematopoietic CD34^+^ stem cells of colon cancer patients, using previously described protocol of expansion and differentiation to monocytes of cord blood-derived CD34^+^ haematopoietic progenitors. Data show that in two-step cultures, the yield of cells was increased approximately 200-fold, and among these cells, up to 60 % of CD14^+^ monocytes were found. They consisted of two subpopulations: CD14^++^CD16^+^ and CD14^+^CD16^−^, at approximately 1:1 ratio, that differed in HLA-DR expression, being higher on the former. No differences in expression of costimulatory molecules were observed, as CD80 was not detected, while CD86 expression was comparable. These CD14^+^ monocytes showed the ability to present recall antigens (PPD, *Candida albicans*) and neoantigens expressed on tumour cells and tumour-derived microvesicles (TMV) to autologous CD3^+^ T cells isolated from the peripheral blood. Monocytes also efficiently presented the immunodominant HER-2/neu_369–377_ peptide (KIFGSLAFL), resulting in the generation of specific cytotoxic CD8^+^ T lymphocytes (CTL). The CD14^++^CD16^+^ subset exhibited enhanced cytotoxicity, though nonsignificant, towards tumour cells in vitro. These observations indicate that generation of monocytes from CD34^+^ stem cells of cancer patients is feasible. To our knowledge, it is the first demonstration of such approach that may open a way to obtain autologous monocytes for alternative forms of adaptive and adoptive cellular immunotherapy of cancer.

## Introduction

Monocytes/macrophages are important players in the host response to the growing tumour, with both enhancing and inhibitory capacities [1–3]. Despite the former, they are still regarded as potential cells that can be used for cellular forms of cancer immunotherapy. Blood monocytes isolated by cytapheresis have been used as a source of effector cytotoxic cells with rather disappointing results [4]. Such strategies require large numbers of monocytes, sometimes their activation, and are based on their direct cytotoxic activity. It is well established that peripheral blood (PB) monocytes exhibit significant cytotoxicity in vitro, which is mediated by some cytokines, for example, surface bound tumour necrosis factor (TNF), and release of reactive nitrogen and oxygen intermediates (RNI and ROI, respectively) [5–7]. Furthermore, PB monocytes can also act as antigen-presenting cells (APC) [8] which may be useful for presentation of tumour-associated antigens (TAA) for the generation of cytotoxic T lymphocytes used in adoptive immunotherapy [9]. Along this way, microvesicles (MV) that are shed by many cells of the body, in particular these rapidly proliferating, are playing an important role in cell to cell communication [10]. We have previously shown that tumour-derived MV (TMV) carry some determinants of the tumour cells and transfer them to monocytes [11]. It is also known that TMV express TAA [12]. Therefore, TMV may be a useful source of neoantigens to be presented to cytotoxic T cells.

Among two main subpopulations of PB monocytes, CD14^++^CD16^−^ and CD14^+^CD16^++^ [13], the latter possess an enhanced antitumour activity, as judged by an increased production of TNF, interleukin (IL)-12, ROI and cytotoxic activity in vitro [14]. However, CD14^+^CD16^++^ cells are the minor population consisting of approximately 5–10 % of total monocytes [13, 15, 16], and nonproliferating cells may be very limited in numbers that are required for adoptive immunotherapy of cancer [4]. We have previously described the protocol for generation of monocytes from cord blood (CB) haematopoietic CD34^+^ progenitors which may give rise to potentially unlimited numbers of monocytes, as up to 1000-fold increase in cells in comparison with the initial inoculum was obtained, and among them up to 60 % of CD14^+^ cells consisting of two novel subsets CD14^++^CD16^+^ and CD14^+^CD16^−^ were found. They differed not only in the CD14 but also in other determinants expression and functional activity [17].

In the present study, the attempts were undertaken to obtain monocytes from bone marrow (BM) haematopoietic CD34^+^ stem cells of patients with colon cancer and to determine their immunophenotype and some functional activities. This paper shows that monocytes with similar characteristics as CB CD34^+^ cell-derived monocytes, consisting of CD14^++^CD16^+^ and CD14^+^CD16^−^ subsets, can be generated and exhibited APC capacity and cytotoxicity against tumour cells.

## Materials and methods

### Bone marrow biopsy and isolation of CD34^+^ cells

Bone marrow from patients with colorectal cancer (Duke’s C stage) was obtained by needle aspiration from the iliac crest, after written consents from the patients. The 15 patients (6 females and 9 males) with mean age 66 ± 14 years, before surgery and without any previous or current treatment were studied. Aspirates were suspended in saline, and mononuclear cells from them and from peripheral blood (PBMC) were isolated accordingly by standard density-gradient centrifugation (Lymphocyte Separation Medium 1077, PAA Laboratories GmbH, Pasching, Austria). Then the CD34^+^ cells were isolated from PBMC using the EasySep Human CD34 Positive Selection Kit (StemCell Technologies, Vancouver, Canada), based on magnetic cell sorting. The mean number of CD34^+^ cells recovered was 1.6 × 10^5^ ± 1.4 × 10^5^. The study was approved by the local Jagiellonian University Ethical Committee (No. KBET/86/B/2007 and KBET/193/B/2011).

### Generation of monocytes

CD34^+^ cells were expanded and differentiated to monocytes in two-step cultures in the expansion and differentiation media, each step 7–10 days, as previously described [17].

### Immunophenotyping

The following anti-human monoclonal antibodies (mAbs) were used: anti-CD14 allophycocyanin (APC)-conjugated, anti-CD16 and anti-HLA-DR, both phycoerythrin (PE)-conjugated, anti-CD80 and anti-CD86, both fluorescein isothiocyanate (FITC)-conjugated (all from BD Pharmingen, San Diego, CA). In parallel, staining with appropriate isotype-matched mouse immunoglobulins (BD Pharmingen) were used as negative controls. After incubation for 30 min at 4 °C with mAbs or isotype controls, the cells were washed, resuspended in 0.3 ml of PBS containing 0.1 % sodium azide and analysed by flow cytometry (FACS Canto, BD Biosciences Immunocytometry Systems, San Jose, CA) using FACS DiVa v. 5.1 software. List mode data for 20,000 events were acquired, and statistical analysis was performed according to the fluorescence intensity of cells stained with appropriate isotype controls.

### Isolation of monocytes and their subpopulations

Cells cultured in the differentiation medium were harvested, washed, and suspended at the concentration of 10 × 10^6^/ml. After staining with anti-CD14 APC and anti-CD16 PE-conjugated mAbs, the cells were sorted using a 100 μm nozzle tip in FACS Aria II (BD Biosciences) into CD14^+^ monocytes (total population) and CD14^++^CD16^+^ and CD14^+^CD16^−^ subpopulations. Sorted cells were collected into polystyrene Falcon 2057 tubes (BD Biosciences) precoated with foetal bovine serum (FBS, Gibco, Paisley, UK), to avoid plastic charging and cell attachment to the wall. The cells were washed and suspended in RPMI 1640 medium (Sigma, St. Louis, MO), supplemented with gentamycin (50 μg/ml), glutamine (2 mM) and 5 % FBS (all from Gibco).

### Isolation of T lymphocytes

The CD3^+^ cells were isolated from PBMC (obtained as above) using the EasySep Human CD3 Positive Selection Kit (StemCell Technologies) based on magnetic cell sorting. Isolated CD3^+^ lymphocytes were suspended in Serum Free Type Cell Freezing Medium (Bambanker, Lymphotec, Tokyo, Japan) and stored at −80 °C until use.

### Tumour cell lines and tumour-derived microvesicles (TMV)

Human pancreatic carcinoma (HPC-4) cell line established in this laboratory [18] and DeTa (colon carcinoma) were cultured and passaged as previously described [19], except that FBS was deprived of MV by centrifugation at 50,000×*g* for 1 h. TMV from HPC-4 cells (TMV_HPC_) were obtained as previously described [11]. Briefly, supernatants from well-grown cell cultures were collected and spun down at 2,000×*g* for 20 min to remove cell debris. Then supernatants were again pelleted (RC28S centrifuge, Sorvall, Newton, CT) at 50,000×*g* for 1 h at 4 °C. Pellets were washed several times in RPMI 1640 to remove FBS and finally resuspended in serum-free RPMI 1640 medium. Quantification of TMV protein concentration was evaluated by the Bradford method (BioRad, Hercules, CA). The cells and TMV_HPC_ were tested for the presence of HER-2/neu using APC-labelled anti-HER-2/neu mAb (BD Biosciences) and flow cytometry analysis (FACS Canto).

### Antigen presentation

The CD34^+^ cell-derived CD14^+^ monocytes (1 × 10^4^/well) isolated by FACS sorting were cultured for 2 h in the presence of recall antigens: purified protein derivative (PPD, 25 μg/ml; Statenserum Institute, Copenhagen, Denmark) or *Candida albicans* (BioRad, Marnes-la-Coqunetté, France), or γ-irradiated (20 Gy) HPC-4 cells, or TMV_HPC_ (5 μg/ml final concentration) or specific TAA antigen–HER2/neu immunodominant peptide KIFGSLAFL (5 μg/ml) in flat-bottom 96-well plates (Sarstedt, Numbrecht, Germany) in RPMI 1640 medium supplemented with l-glutamine (2 mM), 10 % human AB serum and gentamycin (50 μg/ml, all from Gibco). Then, autologous T cells, after thawing and washing three times in RPMI 1640 medium, were added (1 × 10^5^/well). T lymphocytes alone or with the appropriate stimulus and unstimulated cultures were used in parallel as negative controls. Cells were cultured in triplicates for 6 days at 37 °C in 5 % CO_2_ atmosphere, with a 6 h terminal pulse of [^3^H]-thymidine (1 μCi/well). Index of proliferation was calculated according to the formula: cpm of ^3^H-thymidine incorporation in the stimulated culture/cpm of appropriate negative control cultures. BM monocytes were generated, and T lymphocytes were isolated only from the patients whose PBMC proliferated in response to specified stimulants.

### Detection of HER-2/neu-specific cytotoxic CD8^+^ T cells (CTL)

For the detection of HER-2/neu-specific CTL, only patients positive for HLA-A2 antigens were selected. Expression of HLA-A2 was determined by patients’ blood lymphocytes staining, using PE-conjugated mouse anti-human HLA-A2 mAb or PE-conjugated isotype-matched mouse immunoglobulins (both BD Pharmingen) as a negative control, followed by lysis of erythrocytes (FACS Lysing Solution, BD Biosciences) and flow cytometry analysis (FACS Canto). Patients positive for HLA-A2 expression were further tested for the presence of CTL specific to the immunodominant HER-2/neu_369–377_ epitope. For this purpose, whole blood samples were stained with PE-labelled HLA-A*0201 pentamer complex (ProImmune Ltd., Oxford, UK), folded around the HER-2/neu_369–377_-specific epitope. As a negative control, staining with HLA-A*0201 negative control pentamer (ProImmune) was used. The cells were incubated with indicated pentamers for 30 min. at 20 °C followed by washing and staining with peridinin chlorophyll protein complex (PerCP)-conjugated anti-CD3 and FITC-conjugated anti-CD8 mAb (BD Pharmingen) for 30 min. at 4 °C in the dark. Then, the cells were washed and erythrocytes were lysed (FACS Lysing Solution). After additional washing, cells were analysed by flow cytometry (FACS Canto). Data from a minimum 50,000 CD3^+^ cells were collected, and detection of more than 0.2 % HER-2/neu pentamer-stained CD3^+^ CD8^+^ cells above the background was considered positive.

### Generation of HER-2/neu-specific CTL

To check the ability of patients BM CD34^+^ cell-derived monocytes to induce HER-2/neu-specific CTL, the cells from patients positive for primed HER-2/neu_369–377_ CTL were used. T cells were isolated and stored, as described above, until monocytes were generated. After thawing, T cells (5 × 10^5^/well) were cultured with 5 × 10^4^ BM stem cell-derived autologous CD14^+^ monocytes, in the presence of HER-2/neu_369–377_ peptide (KIFGSLAFL; 5 μg/ml, ProImmune) in RPMI 1640 medium, supplemented with l-glutamine (2 mM), 10 % human AB serum and gentamycin (50 μg/ml, all from Gibco). After 7 days of culture, the level of HER-2/neu_369–377_-specific CTL was determined by pentamer staining and flow cytometry analysis, as described above.

### Determination of cytotoxic activity

Cytotoxicity of monocytes and their subpopulations towards HPC-4 and DeTa cells was determined as previously described [6]. Briefly, monocytes (5 × 10^4^/well), tumour cells (2 × 10^4^/well) or their mixtures were cultured in RPMI 1640 medium for 24 h. Then, the culture medium was removed and 100 μl per well of MTT (2 mg/ml; 1, 3-[4,5-dimetylthiazol-2-yl]-2,5-diphenyltetrazolium bromide, Sigma) dye solution was added for 4 h. Formed formazan was extracted with isopropyl alcohol (Fluka Chemie AG, Buchs, Switzerland), containing 0.04 N HCL and its content determined by spectrophotometrical measurement of absorbance using two different wavelengths: 570 and 630 nm. The percentage of cytotoxicity was calculated according to the formula previously described [20]:

$$ \left[ { 1- {\text{OD}}\left( {{\text{monocytes}} + {\text{tumour cells}}} \right) - {\text{OD}}\left( {\text{monocytes alone}} \right)/{\text{OD }}\left( {\text{tumour cells alone}} \right)} \right] \times 100 $$

### Statistical analysis

Nonparametric one-way ANOVA test with the Microcal Origin software v. 5.0 (Northampton, MA) was used for analysis. The differences were considered significant at* p* < 0.05.

## Results

### Immunophenotype of CD34^+^ cell-derived monocytes

**Fig. 1:**
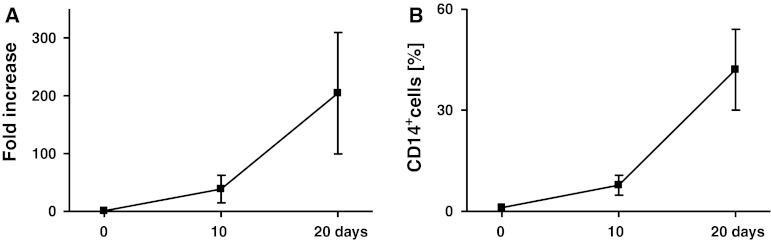
**a** Fold increase in the cell number **b** percentage of CD14^+^ cells during expansion (10 days) and differentiation (10 days) of BM-derived CD34 + cells from colon cancer patients. Mean ± SD from 11 different experiments is shown

**Fig. 2:**
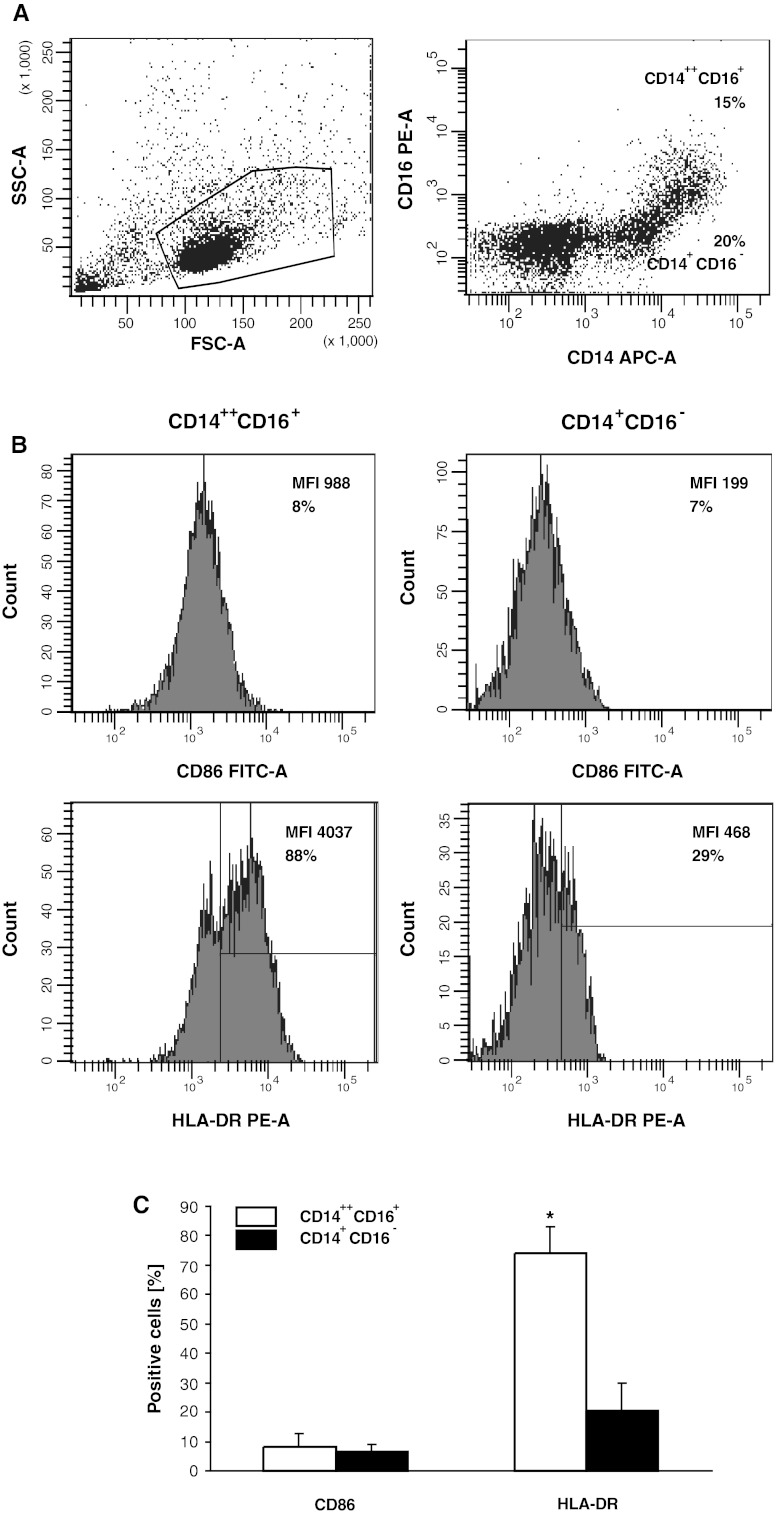
FACS analysis of monocytes generated from BM haematopoietic CD34^+^ stem cells of patients with colon cancer. **a** Morphology according to FSC and SSC parameters (*left*) and expression of CD14 and CD16 determinants (*right*). **b** CD86 and HLA-DR expression on CD14^++^CD16^+^ and CD14^+^CD16^−^ subsets. Markers are set according to the background staining using isotype-matched controls. Representative data from one out of five experiments performed are shown. **c** Mean (± SD) expression of CD86 and HLA-DR on CD14^++^CD16^+^ and CD14^+^CD16^−^ subsets. * denotes significant difference between the subsets

Following culture of CD34^+^ cells in the expansion and differentiation media, the number of cells increased approximately 200-fold (range 75–440) (Fig. 1a) with up to 60 % of CD14^+^ cells at day 20 (Fig. 1b). Among them, CD14^++^CD16^+^ and CD14^+^CD16^−^ subsets in approximately 1:1 proportion were observed (Fig. 2a). Then, the expression of costimulatory molecules on these subsets was determined. The CD80 was not detected, CD86 was comparable on both subsets, while significantly higher HLA-DR expression on CD14^++^CD16^+^ monocytes was observed (Fig. 2b, c).

### Determination of HER-2/neu expression on HPC-4 cells and TMV_HPC_

**Fig. 3:**
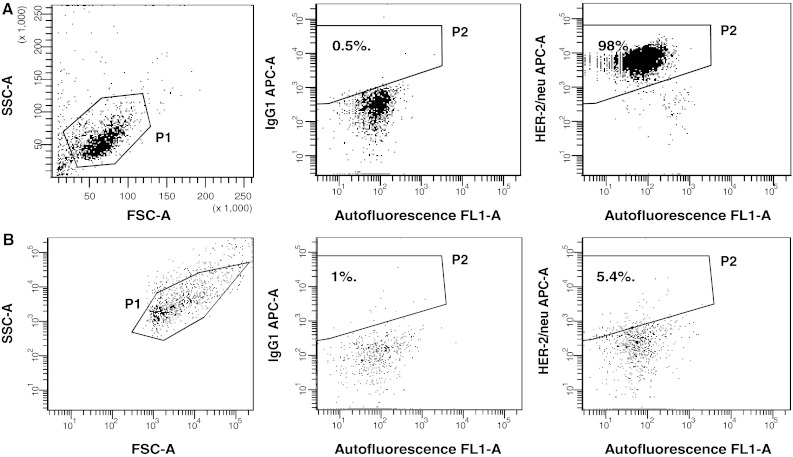
HER-2/neu expression by HPC-4 cells and TMV_HPC_. **a** HPC-4 cells gated according to FSC and SSC parameters—gate P1 (*left*) was stained with APC-conjugated control mouse IgG1 (*middle*) and anti-HER-2/neu mAb (*right*) and analysed by flow cytometry. **b** The expression of HER-2/neu on TMV_HPC_ following staining with the same control isotype and mAb. Gate P2 was set according to staining with isotype-matched control

In order to check whether tumour cells and their TMV used exhibit TAA, expression of HER-2/neu was analysed by flow cytometry. Figure 3a shows that almost all HPC-4 cells expressed HER-2/neu. In contrast, its expression on TMV_HPC_ was markedly lower when compared to the cells they originated from (Fig. 3b). Furthermore, both HPC-4 cells and to a lesser extend TMV_HPC_ expressed MUC-1 and contained HER-2/neu, MAGE-1,3 and MUC-1 mRNA (data not shown). We concluded that both the cells and their TMV may be used as a source of TAA.

### Proliferation of T lymphocytes in response to recall antigens or TAA

**Fig. 4:**
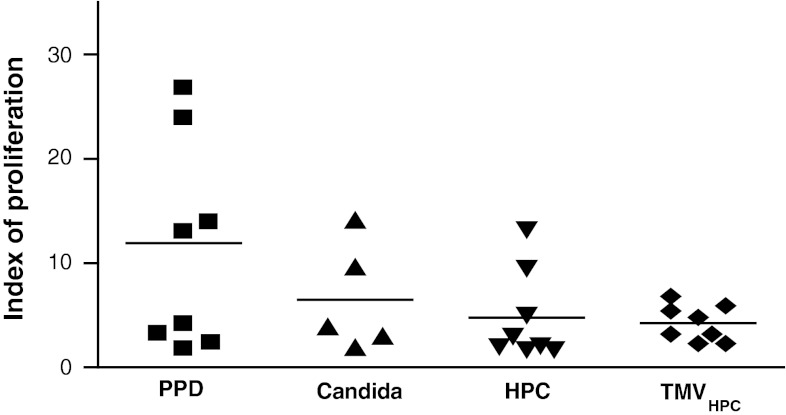
Proliferation of T lymphocytes in the presence of autologous monocytes generated from BM CD34^+^ haematopoietic stem cells of 8 patients with colon cancer. Peripheral blood T lymphocytes were admixed to CD34^+^ cell-derived monocytes either in the medium or preexposed to the stimulants indicated. Results are expressed as the index of proliferation (individual results and mean). Uneven numbers of cultures assessed are due to the response of respective PBMC to the stimuli used. The PBMC from normal subjects did not respond to HPC-4 and TMV_HPC_. Basal (spontaneous) proliferation was 1691 ± 1050 cpm (index 1)

First, to validate the occurrence of T lymphocytes priming, we studied the response of patients’ PBMC to recall antigens (PPD, Candida), γ-irradiated HPC-4 cells or TMV_HPC_. For further studies, only T lymphocytes and BM CD34^+^ cells from patients whose PBMC responded to the stimulants were used. PBMC from 8 control subjects did not respond to HPC-4 and TMV_HPC_ (data not shown). The autologous T lymphocytes isolated from PB of cancer patients were added to CD34^+^ cell-derived CD14^+^ monocytes and preexposed to recall antigens, γ-irradiated HPC-4 cells, and TMV_HPC_. Figure 4 shows that patients’ T lymphocytes responded to recall antigens when cultured with autologous monocytes. The cells also proliferated following stimulation with HER-2/neu-positive HPC-4 cells or TMV_HPC_. T lymphocytes alone cultured in the presence of stimulants did not proliferate. It was concluded that CD34^+^ cell-derived monocytes from cancer patients are able to present recall antigens and TAA to autologous T cells.

### Generation of HER-2/neu_369–377_-specific CTL

**Fig. 5:**
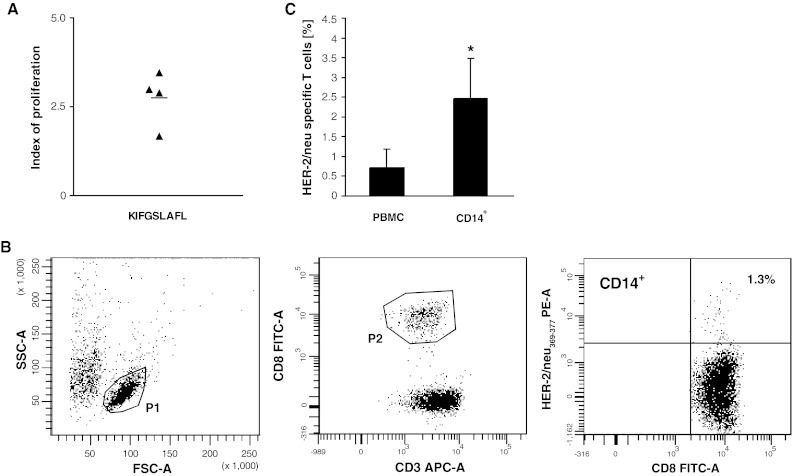
Generation of HER-2/neu-specific CD8^+^ T cells in cultures of BM CD34^+^ cell-derived monocytes with autologous T cells from colon cancer patients, stimulated with HER-2/neu peptide. **a** Proliferation index of T cells in cultures with autologous BM CD34^+^ cell-derived monocytes from colon cancer patients stimulated with HER-2/neu peptide (KIFGSLAFL). **b** Flow cytometry analysis of the level of HER2/neu-specific CTL propagated in the presence of BM CD34^+^ cell-derived monocytes. T cells from HLA-A2-positive patients were cultured with autologous BM CD34^+^ cell-derived CD14^+^ monocytes in the presence of KIFGSLAFL peptide for 7 days, and after staining with PE-conjugated HLA-A*0201 HER-2/neu_369–377_ pentamer and anti-CD3 and anti-CD8 mAbs were analysed by FACS. CD8^+^ T lymphocytes were gated according to morphological (P1) and phenotype marker characteristics (P2). Statistics was set according to staining with HLA-A*0201-negative control pentamer. Data from one representative analysis is shown. The initial level of HER-2/neu_369–377_-specific CTL detected in PBMC of this patient was 0.25 % (not shown). **c** The cumulative data of the initial levels of HER-2/neu_369–377_-specific CTL detected in PBMC of HLA-A2-positive patients and at the end of culture. Data from four performed experiments are shown. * denotes significant difference from PBMC

As the HER-2/neu-derived peptides, naturally processed as TAA, are recognized by tumour-specific, HLA-A2-restricted CTL in colorectal cancer [21], in the next set of experiments we evaluated the proliferative response of T cells stimulated with HER2/neu_369–377_ immunodominant peptide in the presence of patients’ BM CD34^+^ cell-derived monocytes as APC. For this part of study, only patients positive for HLA-A2 expression and for the occurrence of primed CTL specific to the immunodominant HER-2/neu_369–377_ epitope in the blood were selected. This group contained four patients whose level of HER-2/neu-specific CTL in the blood exceeded 0.2 % (range 0.25–1.2 %). T cells isolated from these patients were cultured with population of autologous CD14^+^ BM CD34^+^ cell-derived monocytes and stimulated with HER-2/neu_369–377_ peptide (KIFGSLAFL). After 7 days of culture, proliferation index increased (Fig. 5a). Simultaneously, the level of peptide-specific CTL was determined by flow cytometry (Fig. 5b) and found to be significantly increased in cultures of monocytes with T cells, as compared to PBMC (Fig. 5c). This indicates that BM CD34^+^ cell-derived CD14^+^ monocytes induced propagation of HER2/neu-specific CTL.

### Cytotoxicity

**Fig. 6:**
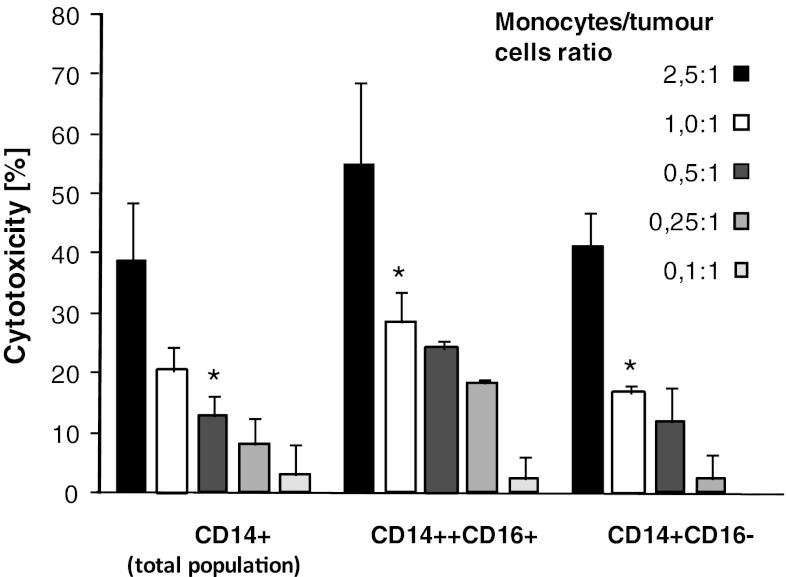
Cytotoxicity of cancer patients’ monocytes and their subsets towards tumour cells. Monocytes were added to tumour cells (HPC-4 or DeTa) at different ratios and cultured for 18 h. Percentage of cytotoxicity, as determined by MTT test, is shown. Mean ± SD from 8 different experiments is indicated. * denotes significantly difference from higher ratio

The total population of monocytes (CD14^+^ cells), used at different doses, exhibited substantial spontaneous cytotoxicity towards HPC-4 and DeTa cancer cells (Fig. 6). The CD14^++^CD16^+^ monocytes showed slightly higher cytotoxic/cytostatic activity in comparison with total population of monocytes or their CD14^+^CD16^−^ subset, though the differences were statistically not significant.

## Discussion

The present observations clearly demonstrate the feasibility of in vitro generation of monocytes from BM CD34^+^ haematopoietic stem cells of colon cancer patients. This model study was limited to a few patients and a small volume of BM aspirates that were available. We have used the protocol that was elaborated for production of monocytes from CB haematopoietic CD34^+^ stem cells, which enabled up to 1000-fold increase in cultured cells among which up to 60 % were CD14^+^ monocytes, and included the two-step cultures of the expansion and differentiation. These monocytes consisted of novel CD14^++^CD16^+^ and CD14^+^CD16^−^ subpopulations with the ratio 1:2 and were clearly different from PB monocytes subsets by phenotype and functions [17]. The use of BM CD34^+^ cells from cancer patients also led to the production of up to 60 % CD14^+^ monocytes containing these subsets, however, occurring at the ratio 1:1. The reason for the differences in the ratios of the monocyte subsets generated from BM and CB CD34^+^ cells are unknown but may be due to the initial kinetic state of these cells or their level of lineage commitment [22]. These subsets showed similar expression of costimulatory molecule CD86, the lack of CD80 and an enhanced expression of HLA-DR detected mostly on CD14^++^CD16^+^ monocytes. The latter finding is similar to this CB monocyte subset, which shows an increased allostimulatory capacity [17] and PB CD14^+^CD16^++^ subpopulation [14]. The increased expression of HLA-DR may imply the higher potential of this subset to act as APC. However, due to a small number of cells available, antigen-presenting ability in respect to recall antigens and TAA was studied only for the total population of obtained monocytes. For determination of APC activity, patients were selected on the basis of their PBMC response to these antigens. The response of autologous T cells cultured with monocytes to recall antigens was observed, which is similar to PB monocytes [8]. Furthermore, such cultures stimulated with tumour cells or TMV that express HER-2/neu and possibly other neoantigens also responded. However, no proliferation was observed in cultures with the addition of patients’ plasma, which is known to contain TMV. This may be due to the relatively small concentration of TMV in the plasma [23]. Also, no response of T cells cultured with autologous CB CD34^+^ cell-derived monocytes, used as control, to the stimuli employed was detected, though these cultures were responding to polyclonal stimulation with anti-CD3 mAb (data not shown). This may indicate that nonprimed CB T lymphocytes did not proliferate in response to TAA. It is also supported by the finding that patients, but not healthy donors PBMC, responded to HPC-4 cells or TMV_HPC_ (data not shown). We wish to suggest that response of patients’ T lymphocytes to HPC-4 cells and TMV_HPC_ is driven by TAA which are expressed on their surface. Our data indicate that HER-2/neu-specific CTL are detected in the blood of colon cancer patients. These CTL were propagated in response to the immunodominant HER-2/neu_369–377_ peptide presented by patients’ autologous BM CD34^+^ cell-derived monocytes. This observation indicates that BM CD34^+^ cell-derived monocytes are potent APC and can be used for expansion of TAA-specific CTL; however, the protocol for more effective yield of expanded CTL needs to be further elaborated as the increase in HER-2/neu_369–377_ CTL number in our study was not impressive when compared to other studies using dendritic cells for stimulation with tumour-derived peptides [24, 25]. It should be noted that we used for their generation short-term cultures and low initial numbers of T cells, which were available for the study. In addition, we are aware of the fact that these cells were determined only phenotypically and no functional studies of CTL were performed, as the number of CTL obtained was very low. These limited observations were initiated only to proof the feasibility of this approach.

The question may arise, whether monocytes obtained from BM-derived CD34^+^ cells of healthy donors behave differently or in the same way as those from cancer patients. Due to ethical reasons, such controls were not introduced into our study. However, the data obtained from CB CD34^+^ cell-derived monocytes suggest that the absence of the response in their cultures with autologous T cells is rather due to the lack of priming the latter and not to the inability of monocytes to act as APC. This observation also indirectly indicates the specificity of the response of patients’ T cells.

It is known that human PB monocytes express spontaneous cytotoxicity towards tumour cells in vitro, which is thought to be associated with their production of TNF, ROI, RNI [6, 14, 26]. Among PB monocytes, CD14^+^CD16^++^ subset (nonclassical monocytes) [13] exhibits a higher cytotoxicity towards tumour cells [14]. The present data indicate that CD14^+^ monocytes generated from BM stem cells of colon cancer patients also show substantial cytotoxicity, which was rather associated (nonsignificantly) with CD14^++^CD16^+^ subset. This makes this subset similar to PB CD14^+^CD16^++^ subpopulation [14]. Therefore, we cannot assign cytotoxicity of generated monocytes to any particular subset. However, we wish to suggest that in general, BM CD34^+^ cell-derived monocytes of cancer patients possess a higher cytotoxic/cytostatic activity, around 40 %, while such monocytes generated from CB around 20 % [27].

In summary, the data presented provide evidence that it is possible to generate monocytes from BM haematopoietic CD34^+^ stem cells of cancer patients, which show the ability to effectively present TAA or HER-2/neu_369–377_ dominant peptide and to act as cytotoxic cells against tumour cells in vitro. It may open the way for generation, potentially on large scale, autologous monocytes that can be used in various forms of adaptive and adoptive cellular immunotherapy of cancer.
